# Supplementary material for: Silica Shell Thickness-Dependent Fluorescence Properties of SiO2@Ag@SiO2@QDs Nanocomposites
Source: Int J Mol Sci. 2022 Sep 2;23(17):10041. doi: 10.3390/ijms231710041 (PMC9456444; doi:10.3390/ijms231710041)
Supplement: Supplementary file 1 [file ijms-23-10041-s001.zip › ijms-1887964-supplementary.pdf]

Supplementary Information

Silica Shell Thickness-Dependent Fluorescence Properties of SiO_2@Ag@SiO_2@QDs Nanocomposites

Eunil Hahm [1], Ahla Jo [1], Sang Hun Lee [2], Homan Kang [3], Xuan-Hung Pham [1] and Bong-Hyun Jun [1,*]

[1] Department of Bioscience and Biotechnology, Konkuk University, Seoul 05029, Korea
[2] Department of Chemical and Biological Engineering, Hanbat National University, Deajeon 34158, Korea
[3] Gordon Center for Medical Imaging, Department of Radiology, Massachusetts General Hospital, Harvard Medical School, Boston, MA 02114, USA
* Correspondence: bjun@konkuk.ac.kr; Tel.: +82-2-450-0521

Keywords: fluorescence; silica shell; fine control; shell thickness; assembled structures; MEF; PRET

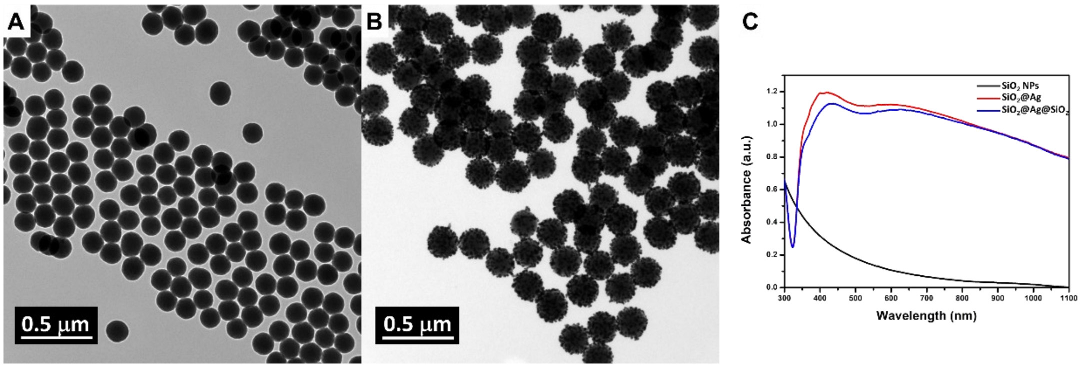

Figure S1. TEM images of (A) SiO_2 NPs and (B) SiO_2@Ag. (C) Absorbance spectra of the SiO_2 NPs, SiO_2@Ag, and typical SiO_2@Ag@SiO_2.

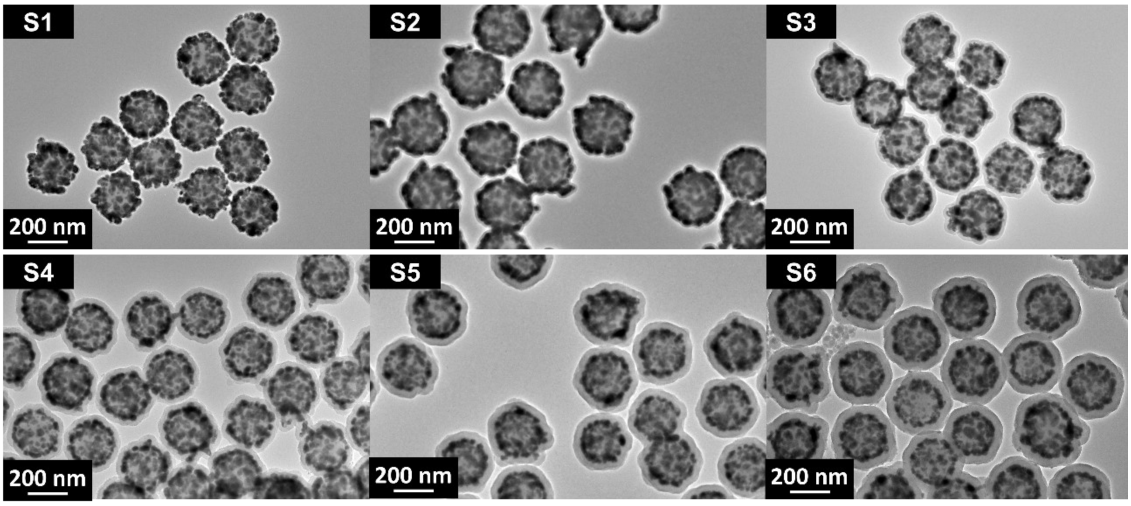

Figure S2. Low-magnification TEM images of SiO_2@Ag@SiO_2 with various silica shell thicknesses on the SiO_2@Ag surface (S1: 4 ± 0.3 nm, S2: 9 ± 1.1 nm, S3: 13 ± 1.2 nm, S4: 16 ± 1.0 nm, S5: 24 ± 1.3 nm, and S6: 38 nm ± 2.0 nm).

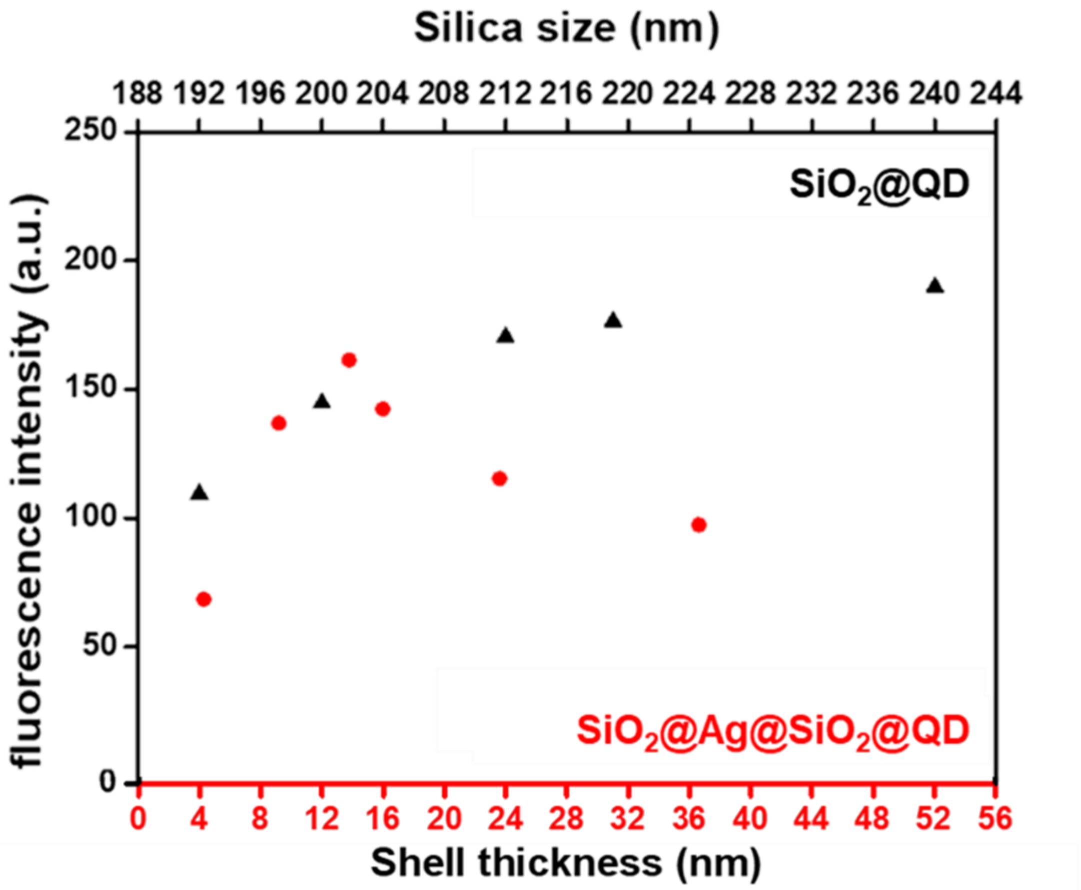

Figure S3. Comparison of the fluorescence properties of the SiO_2@Ag@SiO_2@QDs and SiO_2@QDs.
